# Survival in water of *Campylobacter jejuni* strains isolated from the slaughterhouse

**DOI:** 10.1186/s40064-015-1595-1

**Published:** 2015-12-22

**Authors:** Hana Trigui, Alexandre Thibodeau, Philippe Fravalo, Ann Letellier, Sebastien P. Faucher

**Affiliations:** 1 Department of Natural Resource Sciences, Faculty of Agricultural and Environmental Sciences, McGill University, 21,111 Lakeshore Road, Ste-Anne-de-Bellevue, Montreal, QC H9X 3V9 Canada; 2 Department of Pathology and Microbiology, University of Montreeal, Saint-Hyacinthe, QC Canada

## Abstract

*Campylobacter jejuni* cause gastroenteritis in humans. The main transmission vector is the consumption or handling of contaminated chicken meat, since chicken can be colonized asymptomatically by *C. jejuni*. However, water has been implicated as the transmission vector in a few outbreaks. One possibility is the contamination of water effluent by *C. jejuni* originating from chicken farm. The ability of *C. jejuni* to be transmitted by water would be closely associated to its ability to survive in water. Therefore, in this study, we have evaluated the ability of reference strains and chicken-isolated strains to survive in water. Defined water media were used, since the composition of tap water is variable. We showed that some isolates survive better than others in defined freshwater (Fraquil) and that the survival was affected by temperature and the concentration of NaCl. By comparing the ability of *C. jejuni* to survive in water with other phenotypic properties previously tested, we showed that the ability to survive in water was negatively correlated with autoagglutination. Our data showed that not all chicken isolates have the same ability to survive in water, which is probably due to difference in genetic content.

## Background

*Campylobacter* is the main cause of bacterial food-borne infection in industrialized countries (Dasti et al. 2010). *Campylobacter* infection is characterized by the colonization of the lower intestine by the bacterium, which causes symptoms including fever, abdominal cramps and diarrhea (Dasti et al. 2010; Epps et al. 2013). Severe cases are associated with complications, such as the Guillain Barré Syndrome (Dasti et al. 2010; Epps et al. 2013). The annual incidence of campylobacteriosis in Canada in 2010 was 26.3 cases per 100,000 persons, being relatively stable since 2006 (Public Health Agency of Canada 2014). It is estimated that the annual costs associated with this disease in the USA is approximately $1.7 billions (Batz et al. 2011).

*Campylobacter jejuni* and *Campylobacter coli* are responsible for about 90 % of campylobacteriosis in humans (Dasti et al. 2010; Bolton 2015). *C. jejuni* is commonly found in the gastrointestinal tract of broiler chicken and wild birds, while *C. coli* is usually more prevalent in other animals (Dasti et al. 2010; Epps et al. 2013). Most cases are due to the consumption or handling of poultry, raw milk and untreated water (Wilson et al. 2008; Dasti et al. 2010; Epps et al. 2013). While outbreaks of *Campylobacter* occur occasionally, most cases are sporadic. A multi-locus sequence typing study reveals that 97 % of sporadic cases are due to strains with an animal origin, such as chicken, cattle and sheep, while only 3 % are caused by environmental strains (Wilson et al. 2008). Nevertheless, the mode of transmission of animal strains is not necessary always the consumption of contaminated animal product such as meat and raw milk. Indeed, there are many accounts of campylobacteriosis outbreaks caused by the consumption of drinking water (Vogt et al. 1982; Lind et al. 1996; Clark et al. 2003; Kuusi et al. 2004; O’Reilly et al. 2007). In some of these outbreaks, the drinking water was pumped from groundwater wells, lakes or rivers, which were likely contaminated with livestock manure coming from neighboring farms (Vogt et al. 1982; Clark et al. 2003) and/or with sewage (Vogt et al. 1982; Lind et al. 1996; O’Reilly et al. 2007). Indeed, the incidence of *Campylobacter* infection is tightly correlated with the load of *Campylobacter* in sewage effluent (Jones 2001). In addition, incidence of *Campylobacter* infection peaks at the end of the spring presumably caused by cyclical variation in livestock carriage of *Campylobacter* (Jones 2001). Therefore, proximity to the livestock reservoir could be a risk factor for human infection. Lévesque et al. (2013) have performed a prospective study of the source of sporadic cases in urban and rural area in Quebec. It was found that inhabitants of rural area have a 1.89 fold higher risks of contracting campylobacteriosis than inhabitants of urban area (Lévesque et al. 2013). Moreover, they found that the two most important risk factors in rural area were the occupational exposure to animals, and the consumption of water from a private well (Lévesque et al. 2013). Taken together, these observations suggest that the contamination of drinking water supplies with strains of animal origin is an important mode of transmission for campylobacteriosis (Bronowski et al. 2014).

Survival of *Campylobacter* in water is therefore critical for the transmission to humans trough the consumption of contaminated drinking water and for the transmission from one animal reservoir to another (Bronowski et al. 2014). Many factors influence the survival of *Campylobacter* in water such as temperature, concentration of dissolved organic matters, and dissolved minerals (Buswell et al. 1998; Cools et al. 2003; Baffone et al. 2006; Tatchou-Nyamsi-König et al. 2007; 2008). Studies reporting the survival of *Campylobacter* in water have used different water, such as tap water (Buswell et al. 1998; Cools et al. 2003), bottled mineral water (Tatchou-Nyamsi-König et al. 2007) and artificial seawater (ASW) medium (Baffone et al. 2006). Interestingly, the origin of the strains seem to influence the survival in water; chicken isolates surviving better than clinical isolates (Buswell et al. 1998; Cools et al. 2003). Nonetheless, there is a huge variability in the survival of strains of clinical origin in ASW (Baffone et al. 2006). It was also shown that *Campylobacter* could enter a viable but non-culturable (VBNC) state after prolonged exposure to water (reviewed in Bronowski et al. 2014; Li et al. 2014). Baffone et al. (2006) showed that clinical isolates in the VBNC state can be resuscitated by passage in the mouse intestine (Baffone et al. 2006).

We have recently isolated strains from chicken caecal contents at the time of slaughter. Since survival in water can be an important determinant of *Campylobacter* ability to cause water-borne outbreaks and sporadic cases, we sought to determine the survival of these *C. jejuni* chicken-isolated strains in water. We choose to use artificial water medium (Fraquil, ASW and Fraquil-SALT) to alleviate the variability of tap water composition.

## Results and discussion

### General study design

The survival of 9 isolates of *C. jejuni* was tested in artificial water medium. Two isolates are reference strains NCTC11168 and RM1221. NCTC11168 was isolated in 1977 from a case of human infection (Gaynor et al. 2004). These isolates seems to have a lower ability to colonize the chicken then other isolates (Ahmed et al. 2002). RM1221 was isolated from store-bought chicken meat (Miller et al. 2000). The remaining strains were isolated from chicken caecal contents at the time of slaughter in a slaughterhouse located in Quebec, Canada, as part of previously published studies (Thibodeau et al. 2013, 2015). The survival of the 9 strains was evaluated in a freshwater medium (Fraquil), in artificial seawater medium (ASW), and in Fraquil supplemented with 2.6 % NaCl (Fraquil-Salt).

### Effect of temperature and survival in artificial freshwater medium (Fraquil)

**Fig. 1:**
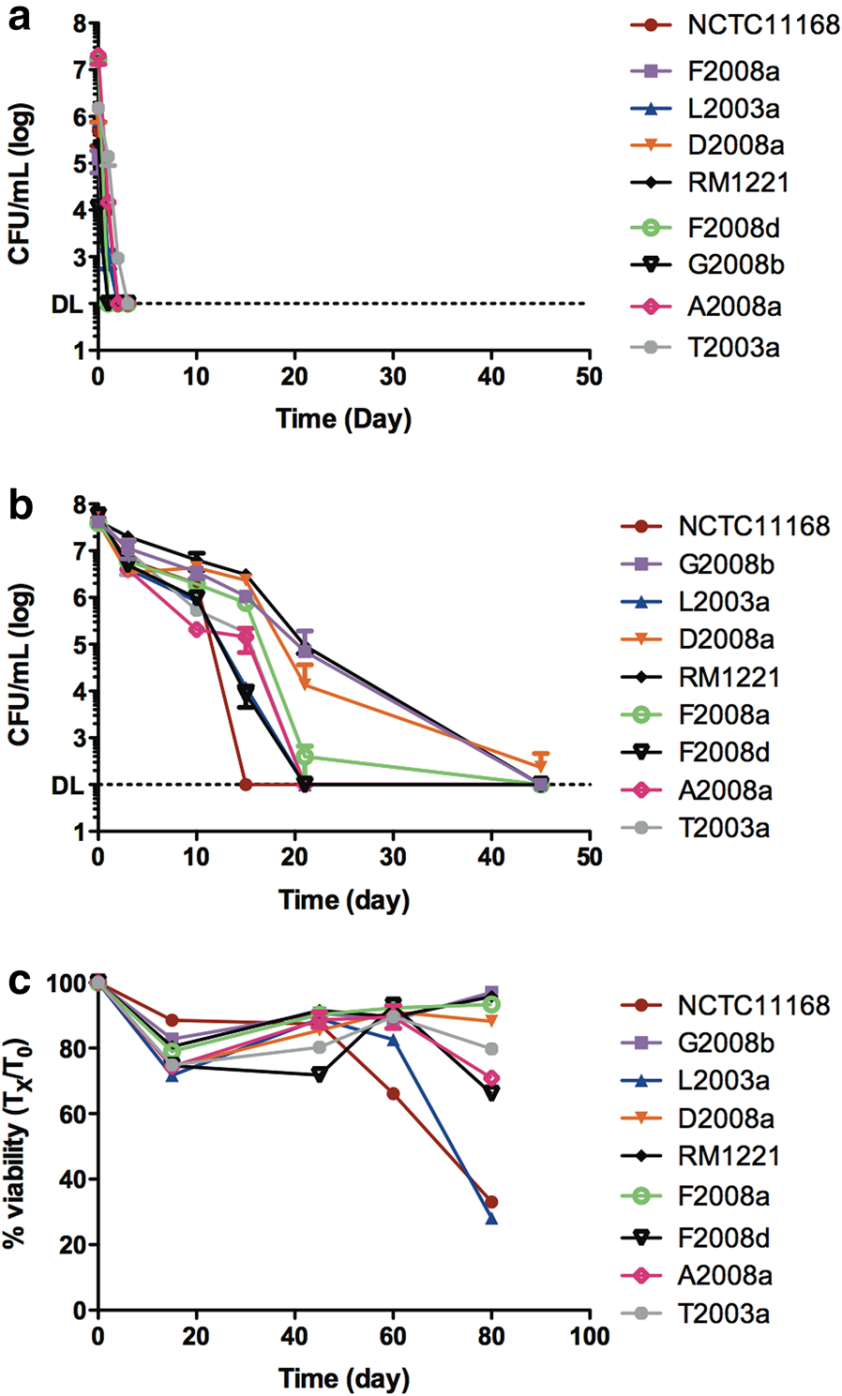
Survival of *C. jejuni* in Fraquil. Strains of *C. jejuni* were suspended in Fraquil at an OD_600_ of 0.1 and incubated at 25 °C (**a**) and 4 °C (**b**, **c**). The survival was monitored by CFU counts on TSA-blood (**a**, **b**). The viability at 4 °C was determined with a Live/Dead stain and flow cytometry (**c**)

**Fig. 2:**
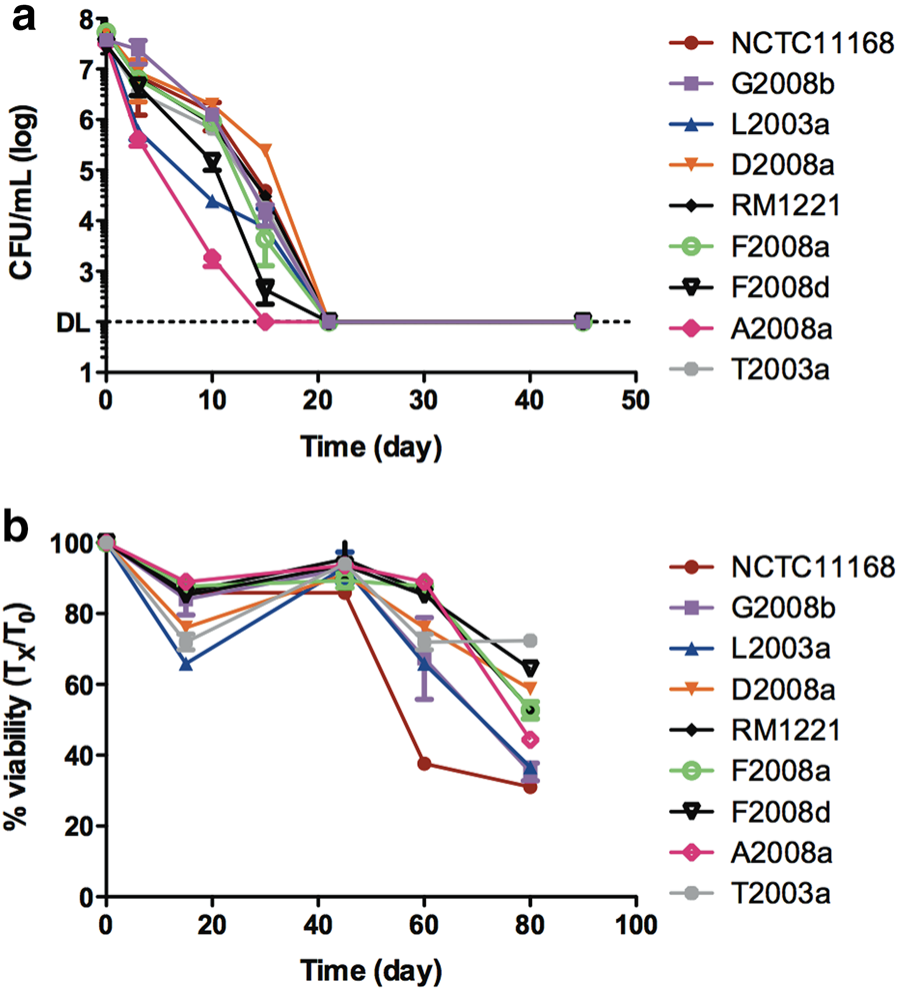
Survival of *C. jejuni* in ASW. Strains of *C. jejuni* were suspended in ASW at an OD_600_ of 0.1 and incubated 4 °C. The survival was monitored by CFU counts on TSA-blood (**a**). The viability was determined with a Live/Dead stain and flow cytometry (**b**)

Temperature is an important factor influencing the survival of *C. jejuni* in water (Bronowski et al. 2014). Cold temperature of 4 °C favors survival, whereas a temperature of 25 °C and higher is detrimental (Buswell et al. 1998; Thomas et al. 1999; Tatchou-Nyamsi-König et al. 2007). Since we are the first to evaluate the survival of *C. jejuni* in Fraquil, we first confirmed the effect of temperature seen in other study. The strains were therefore suspended in Fraquil and incubated at 4 °C or 25 °C. After 3 days at 25 °C, the CFU of all strains was reduced to the detection limit, less than 100 CFU ml^−1^ (Fig. 1a). In contrast, the CFU counts of the samples incubated at 4 °C showed a slow decline, reaching the detection limit about 3–4 weeks later (Fig. 2b). Our results are comparable to other studies showing that *C. jejuni* is less tolerant to warm temperatures (Buswell et al. 1998; Thomas et al. 1999; Talibart et al. 2000; Tatchou-Nyamsi-König et al. 2007). There was a great variability in the survivorship of the different strains at 4 °C. After 21 days, strain G2008b, D2008a and RM1221, showed CFU counts of approximately 10^5^ per mL, whereas the CFU counts of NCTC11168, F2008a, A2008a, L2003a, F2008d, and T2003a, were close to or had reached the detection limit. The viability of each strain was also monitored over time to detect the presence of potential VBNC form. We used the Live/Dead BactLight kit (Invitrogen). This kit contains two dyes, Syto 9 and propidium iodide. Syto 9 stains all types of cells, whereas propidium iodide stains only cells with membrane damage, an indication that the cells are dead (Li et al. 2014). This kit has been used to determine the viability of *C. jejuni* using microscopy (Cameron et al. 2012; Ghaffar et al. 2015), but the authors found that the PI stain is somewhat unreliable. To circumvent this limitation, we used flow cytometry to analyse a large proportion of cells and perform calibration by using a fresh suspension of *C. jejuni* in Fraquil (live control) and an aliquot of this suspension heated at 100 °C for 10 min (dead control). After 80 days in water, most strains still showed viability higher than 60 % (Fig. 1c). Only strains NCTC11168 and strain L2003a showed a lower viability of around 30 %. Nonetheless, this shows that a large number of cells (e.g. 30 % of 5 × 10^7^ per mL) were in a VBNC form after 80 days of incubation.

The survival of the reference strains NCTC11168 and RM1221 was consistent with previous study showing that chicken isolates survived better in water than clinical isolates (Buswell et al. 1998; Cools et al. 2003). Indeed, the CFU counts of RM1221 were higher than NCTC11168, and it took longer for RM1221 to reach the detection limits. Moreover, the viability of NCTC11168 after 80 days of incubation was also lower than RM1221. However, only two of our chicken isolates (G2008b and D2008a) were as good at surviving in water as RM1221, the others were similar to NCTC11168. Our data seem to contradict the notion that chicken isolates survive better than clinical isolates. Our data shows that the survivability in water trait is quite variable, and does not correlate with the origin of strains. Similar variability in this trait was reported before (Talibart et al. 2000). Our chicken isolates were harvested at the time of slaughter, whereas RM1221 was isolated from chicken meat from the grocery store. It is not clear at what stage the chicken isolates used in Buswell et al. 1998 and Cools et al. (2003) were collected. It is possible that some processes in the slaughterhouses, or simply the ability to survive on chicken carcasses, could select for strains that survive better in water. Indeed, the population of *Campylobacter* present on chicken carcasses is different than the population found in chicken ceacal content (Normand et al. 2008; Bily et al. 2010; Colles et al. 2010; Kudirkienė et al. 2011). Presumably, the cooling water tanks are a key environment for the dispersion and selection of strains (Kameyama et al. 2012). Therefore, it can be postulated that at the time of slaughter, the chicken would harbor different types of strains (Rivoal et al. 1999), some surviving well in water, and some surviving poorly; however, strains collected on the chicken carcasses will all have good ability to survive in water. We are planning to study this possibility further.

### Survival in artificial seawater

Baffone et al. (2006) have used artificial seawater medium (ASW) to evaluate the survival of clinical isolates. We were curious to see how well our isolates survive in this medium. Therefore, our two model strains and our isolates were suspended in ASW and incubated at 4 °C. Then the CFU counts and the viability were monitored as described above. In general, the strains reached the detection limit quicker in ASW than in Fraquil (compare Figs. 1b, 2a). The reference strains NCTC11168 and RM1221 had a similar survival pattern, both reaching the detection limit after 21 days of incubation. The viability was also lower in ASW after 80 days of incubation than in Fraquil. Our results are consistent with Baffone et al. (2006) in which most of the strains studied were not countable after 25 days of incubation in ASW.

### Survival in Fraquil-Salt

**Fig. 3:**
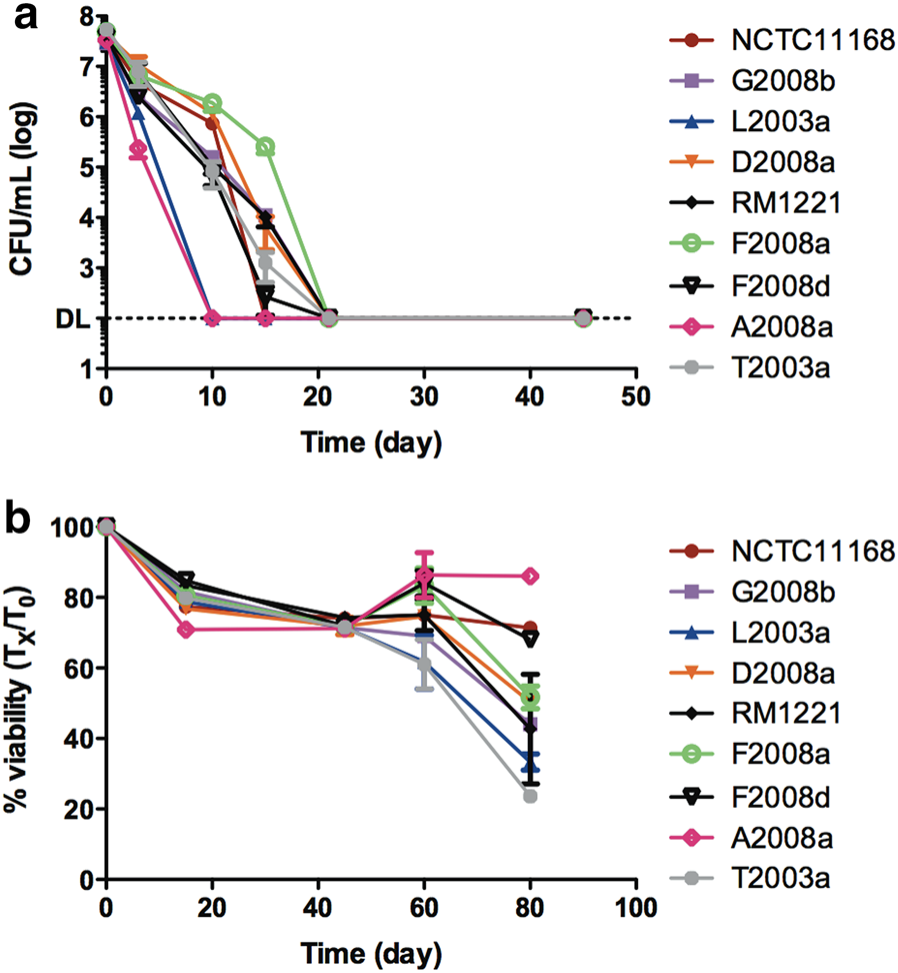
Survival of *C. jejuni* in Fraquil-Salt. Strains of *C. jejuni* were suspended in Fraquil-Salt at an OD_600_ of 0.1 and incubated 4 °C. The survival was monitored by CFU counts on TSA-blood (**a**). The viability was determined with a Live/Dead stain and flow cytometry (**b**)

Since the composition of ASW is quite different than the composition of Fraquil, we sought to determine whether the difference in survival was mostly due to the high concentration of NaCl of ASW or to other components. Therefore, our strains were suspended in Fraquil supplemented with 2.6 % NaCl, the concentration of NaCl found in ASW. The suspensions were incubated at 4 °C and the counts and viability were determined as described above. In general, the strains showed a similar reduction in CFU per mL and in viability in Fraquil-Salt than in ASW (Figs. 2a, 3a). This indicates that the higher concentration of NaCl is detrimental to the survival of *C. jejuni* in ASW. Indeed, it was shown that motility and growth of *C. jejuni* were significantly impaired at or above 2 % NaCl (Cameron et al. 2012).

### Association between survival in water and other traits

**Fig. 4:**
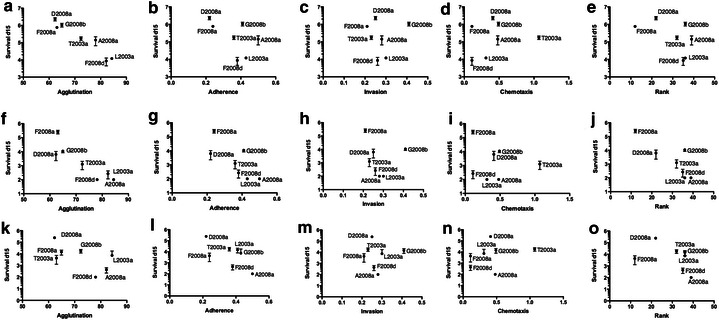
Correlation between the survival in Fraquil (**a**–**e**), Fraquil-Salt (**f**-**j**) and ASW (**k**–**o**) and 5 phenotypes previously tested for those strains: agglutination, adherence, invasion, chemotaxis, and overall rank (Thibodeau et al. 2015)

The chicken isolates were previously tested for many phenotypes associated with colonization of the chicken gut, including autoagglutination, adherence to and invasion of primary caecal cells, and chemotaxis (Thibodeau et al. 2015). The results of these tests were used to calculate an overall rank for each strain. In three different trials, high rank strains were shown to outcompete lower rank strains during co-colonization of chicken. Therefore, strains with a higher rank seem to better colonize chicken than other strains (Thibodeau et al. 2015). We have tried to establish correlation between each of those phenotypes and the survival of each isolates after 15 days in Fraquil, Fraquil-Salt, and ASW (Fig. 4). No correlation was detected between rank and survival in water (Fig. 4e, j, o). Of note, G2008b was shown to outcompete low-rank strain during co-colonization of chicken (Thibodeau et al. 2015), and is one of the best strains at surviving in water (Fig. 1b). At the opposite, D2008a survived well in water but had a low rank, and was outcompeted by a higher rank strain (Thibodeau et al. 2015). Some high-ranking strain, such as F2008d, which outcompeted D2008a, showed a poor survivability in water (Thibodeau et al. 2015). Therefore, better adaptation to the chicken environment does not necessary imply a good survivorship in water.

In addition, we found a statistically significant negative correlation between autoagglutination and survival in Fraquil (*P* = 0.0067) and Fraquil-Salt (*P* = 0.035). Autoagglutination could lead to the sedimentation of *C. jejuni* at the bottom of the tube and biases the sampling, and therefore lower the CFU counts of strains showing high autoagglutination. Since the tubes were inverted multiple times before sampling, this explanation is rather unlikely. One possibility is that agglutination and survival in water are incompatible, e.g. higher agglutination leads to lower survival. For example, expression of genes associated with autoagglutination could reduce the survival in water. There are a few genes known to be involved in autoagglutination, including genes involves in motility (Golden and Acheson, 2002), flagellin glycosylation (Guerry et al. 2006; van Alphen et al. 2008), and the carbon starvation protein A gene (cstA) (Rasmussen et al. 2013).

## Conclusion

In this study we have investigated the survival of *C. jejuni* chicken cecal isolates in Fraquil, ASW and Fraquil-Nacl. There seems to be a great variability in the survivability of the different strains in Fraquil, which mimics freshwater. Our data suggest that some chicken isolates have a greater potential at being transmitted by water than others. Survival in water seems to be inversely correlated with autoagglutination. Difference in the genetic content between the strains could explain this variability.

## Methods

### Strains and growth media

*Campylobacter jejuni* reference strains RM1221 and NCTC11168 (ATCC 700819) were acquired from Cedarlane (Ontario, Canada). The other strains were isolated from chicken at the time of slaughter in slaughterhouse located in Quebec, Canada, as part of a previously published study (Thibodeau et al. 2013, Table 1). *C. jejuni* strains were routinely grown on tryptic soy agar (TSA) supplemented with 5 % defibrinated sheep blood (TSA-blood). The plates were incubated at 42 °C in a microaerophillic atmosphere generated with the CampyGen system (Oxoid).

**Table 1:** *Campylobacter jejuni* strains used in this study

Name	Origin	Condition of isolation	Reference
NCTC11168	Human	Clinical isolate	Ahmed et al. (2002)
RM1221	Chicken	Store-bought chicken carcass	Miller et al. (2000)
G2008b	Chicken	Caecal content at time of slaughter	Thibodeau et al. (2015)
L2003a	Chicken	Caecal content at time of slaughter	Thibodeau et al. (2015)
D2008a	Chicken	Caecal content at time of slaughter	Thibodeau et al. (2015)
F2008a	Chicken	Caecal content at time of slaughter	Thibodeau et al. (2015)
F2008d	Chicken	Caecal content at time of slaughter	Thibodeau et al. (2015)
A2008a	Chicken	Caecal content at time of slaughter	Thibodeau et al. (2015)
T2003a	Chicken	Caecal content at time of slaughter	Thibodeau et al. (2015)

### Survival in water

The survival of *C. jejuni* strains was evaluated in three kinds of artificial water media: Fraquil, ASW and Fraquil-Salt. The composition of Fraquil is an approximation of freshwater (0.004 % CaCl_2_, 0.004 % MgSO_4_, 0.001 % NaHCO_3_, 0.0002 % K_2_HPO_4_, 0.004 % NaNO_3_, 10 nM FeCl_3_, 1 nM CuSO_4_, 0.22 nM (NH_4_)_6_Mo_7_O_24_, 2.5 nM CoCl_2_, 23 nM MnCl_2_ et 4 nM ZnSO_4_). Fraquil-Salt is Fraquil supplemented with 2.65 % NaCl. The ASW medium mimics seawater composition (2.65 % NaCl, 0.0725 % KCl, 0.244 % MgCl_2_, 0.114 % CaCl_2_, 0.33 % MgSO_4_, 0.0202 % NaHCO_3_, 0.0083 % NaBr (Baffone et al. 2006). *C. jejuni* strains were grown on TSA-blood for 2 days at 42 °C under microaerophilic atmosphere. A few colonies were collected and suspended in defined water medium, washed three times with the medium and suspended in 5 ml of fresh medium at a final OD_600_ of 0.1 in a 5 ml plastic tube (Sarstedt). The number of viable bacteria was measured over by performing CFU counts on TSA-blood as described above. Fresh medium was used to perform the dilution for the CFU counts. All experiments were performed on at least three biological replicates. The error bars represent standard deviation from the mean.

### Live/Dead staining

The presence of VBNC form of *C. jejuni* following exposure to water was determined by using the Live/Dead BactLight Kit (Invitrogen). At each time point, an aliquot was removed, diluted tenfold in fresh medium, and stained as described by the manufacturer. The stained cells were then counted by flow cytometry using a Guava easyCyte (Millipore). A live control and a dead control were used to setup the region associated with the live population and the dead population. A fresh suspension of *C. jejuni* NCTC1168 in defined water medium was prepared and split in two aliquot. One was used as is for the live control; the other was exposed to boiling water for 5 min to kill the cells and serve as the dead control.

### Statistical analysis

Correlation between survival in water and the phenotypes previously studied (Thibodeau et al. 2015) was assessed using a two-tailed Spearman correlation.
